# IAPV-Induced Paralytic Symptoms Associated with Tachypnea via Impaired Tracheal System Function

**DOI:** 10.3390/ijms221810078

**Published:** 2021-09-17

**Authors:** Yanchun Deng, Sa Yang, Hongxia Zhao, Qingyun Diao, Chunsheng Hou

**Affiliations:** 1 Institute of Apicultural Research, Chinese Academy of Agricultural Sciences, Beijing 100093, China; 18852861125@163.com (Y.D.); sayang1994@163.com (S.Y.); dqyun1@126.com (Q.D.); 2 Key Laboratory of Pollinating Insect Biology, Ministry of Agriculture and Rural Affairs, Beijing 100093, China; 3 Graduate School of Chinese Academy of Agricultural Sciences, Beijing 100081, China; 4 Guangdong Key Laboratory of Animal Conservation and Resource Utilization, Guangdong Public Laboratory of Wild Animal Conservation and Utilization, Institute of Zoology, Guangdong Academy of Sciences, Guangzhou 510260, China; hxzh110@126.com; 5 Institute of Bast Fibre Crops, Chinese Academy of Agricultural Sciences, Changsha 410205, China

**Keywords:** paralytic symptoms, respiratory failure, tissue tropism, IAPV, honey bee

## Abstract

Although it had been reported that Israeli acute paralysis virus (IAPV) can cause systemic infection in honey bees, little is known about how it establishes this infection and results in the typical symptoms, paralysis and trembling. Here, we used our previously constructed IAPV infectious clone to investigate viral loads in different tissues of honey bees and further identify the relation between tissue tropism and paralytic symptoms. Our results showed that tracheae showed a greater concentration of viral abundance than other tissues. The abundance of viral protein in the tracheae was positively associated with viral titers, and was further confirmed by immunological and ultrastructural evidence. Furthermore, higher viral loads in tracheae induced remarkable down-regulation of succinate dehydrogenase and cytochrome c oxidase genes, and progressed to causing respiratory failure of honey bees, resulting in the appearance of typical symptoms, paralysis and body trembling. Our results showed that paralysis symptoms or trembling was actually to mitigate tachypnea induced by IAPV infection due to the impairment of honey bee tracheae, and revealed a direct causal link between paralysis symptoms and tissue tropism. These findings provide new insights into the understanding of the underlying mechanism of paralysis symptoms of honey bees after viral infection and have implications for viral disease prevention and specific therapeutics in practice.

## 1. Introduction

Honey bees provide pollination services for crops and wild plants in both natural and agricultural ecosystems [1]. However, several reports have shown that both managed and wild bee populations are undergoing colony decline in North America and Europe, most likely as a consequence of multiple factors combined, including pollution, pesticide exposure and pathogens [2]. The essential traits of honey bee foraging mean that they are the hosts of numerous parasites and pathogens that can cause severe impacts on colony population growth [3]. Especially, viral infection was considered as the most key player in colony decline due to covert infection most of the time [4]. In addition, the interaction between *Varroa destructor* and the virus not only reduced host immunity but also elevated the level of the virus and led to a rapid decline in colony populations [5]. It has been reported that *V. destructor* is an effective vector of Israeli acute paralysis virus (IAPV) [6], which was found to be the leading cause of colony collapse disorder (CCD) and honey bee mortality worldwide, even if subsequent studies could not confirm the direct link between IAPV and CCD [7].

IAPV is positive single-strand RNA virus belonging to the family Dicistroviridae that is distributed globally [8]. IAPV was identified first in honey bees with typical paralysis symptoms in Israel [9], and then it was found that IAPV had a global prevalence in managed honey bee colonies [10]. Subsequently, it has been found that IAPV can establish systemic infection in honey bees [11] and bumblebees [12]. In addition, several honey bee and wild bee species [12] were found to be infected by IAPV, and even ants [13].

IAPV infection induces a broad spectrum of clinical signs, from asymptomatic infection to a series of apparent depressive symptoms, including paralysis and body trembling, indicating that the virus most likely infects the neuron system [14]. However, recent studies showed that nerve tissues did not harbor the highest viral loads [10]. Transcriptomic analysis was applied on bees with IAPV infection and it was found that the preferred tropism was the gut, followed by nerves [10], while this was contradicted by a recent observation that the fat body harbored the highest level of IAPV, followed by the ovary, brain and midgut [14]. Thus, the relationship between paralysis symptoms and tropism has not been fully determined, hindering our understanding of pathogenesis of IAPV infection and transmission routes. Usually, there are two ways to study viral infection under experimental conditions. One is feeding, which is a major method to study systemic infection. In this case, the digestive tract or gut will induce the local immune response to determine whether the viral infection can pass through the gut [15]. The other is injection to investigate the host response [16]. However, both methods need to fulfill a first requisite that which is to find a tropism to facilitate replication. Thus, a significant roadblock to a detailed understanding of this virus is the lack of a direct link between paralysis symptoms and tissue tropism. These results raise the issue of paralysis symptoms and tissue tropism that needs to be investigated further.

Except for a few viruses, such as sacbrood virus and paralytic viruses including IAPV, most other honey bee viruses frequently cause covert infection without apparent clinical signs [17]. Asymptomatic infection can become symptomatic infection under certain conditions. For example, *Varroa destructor*, an important ectoparasitic mite of honey bees, can vector several bee viruses, including deformed wing virus (DWV) and IAPV, and turn covert infection into overt infection, with typical signs ranging from discoloration, black abdomen, hairlessness and deformed wings to leg paralysis, body trembling and increased mortality [14,18]. In addition, when honey bees are infected with CBPV, acute high doses of pesticide such as imidacloprid will also induce observable symptoms [19]. However, for DWV and IAPV infection, clinical symptoms were not closely linked to the tissue tropism, indicating that overt infection might be associated with the most heavily damaged organs.

To address these gaps in the knowledge, we used an IAPV infectious clone to avoid the potential interference of other viruses to explore the exact tissue tropism [20], and then identified the underlying cause of the typical paralysis signs of IAPV. We found that the trachea was the preferred tissue of IAPV infection and down-regulation of the expression of succinate dehydrogenase (SDH) and cytochrome c oxidase (COX) genes, leading to heavy breathing and progressing to paralysis and trembling. These results showed that paralytic symptoms were not really paralysis but a way of mitigating the shortness of breath.

## 2. Results and Discussion

**Figure 1:**
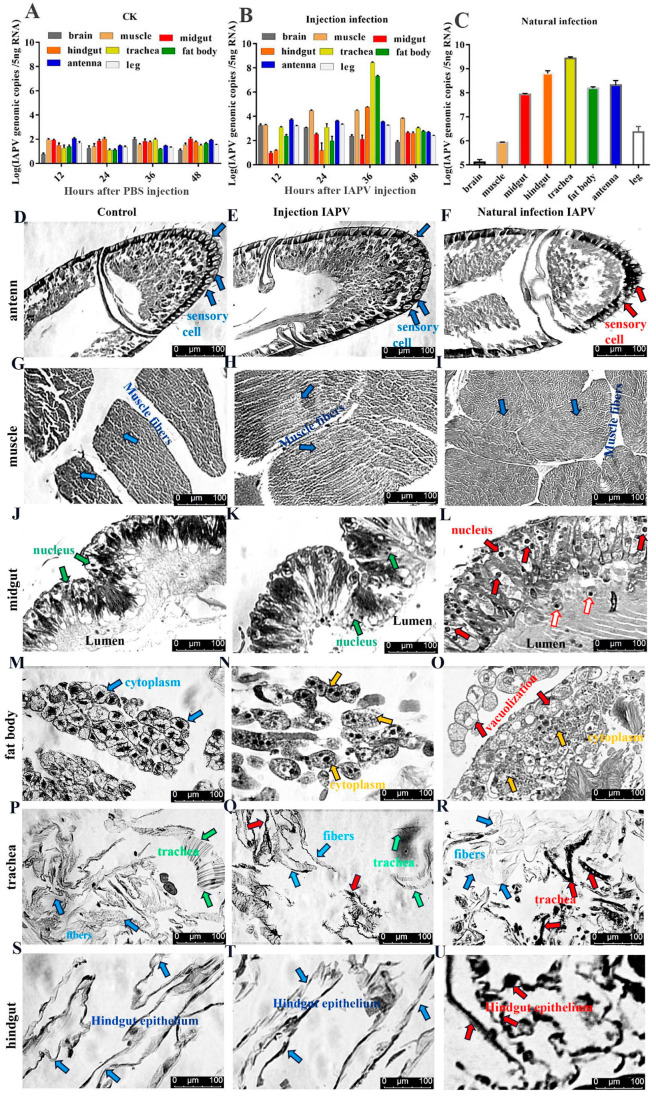
Viral titers in different tissues of bees injected with infectious clone of IAPV at different time points and natural infection, predominantly with IAPV, and the damage of IAPV infection to different tissues. Quantification of IAPV viral titers in trachea, fat body, hindgut, muscle, antenna, brain, midgut and leg tissues of bees from control group (**A**), group infected with IAPV infectious clone at 4 time points (**B**) and group naturally infected, predominantly with IAPV, in the field (**C**). (**D**,**G**,**J**,**M**,**P**,**S**): antenna, muscle, trachea, midgut, fat body and hindgut of control bee group, respectively; (**E**,**H**,**K**,**N**,**Q**,**T**): antenna, muscle, trachea, midgut, fat body and hindgut of bees infected with IAPV infectious clone, respectively; (**F**,**I**,**L**,**O**,**R**,**U**): antenna, muscle, trachea, midgut, fat body and hindgut of bees infected predominantly with IAPV under natural conditions. Arrows point to the tissues that were severely damaged by IAPV infection (red in (**F**): damaged sensory cell; red in (**L**): intestinal epithelial cell with nucleus enlargement; yellow in (**N**): heterogeneous cytoplasm; red in (**O**): vacuolization; red in (**R**): damaged and darkened trachea; red in (**U**): darkened and undermined hindgut; blue and green: tissue with no obvious disease). The tissue sections were observed under 200× magnification.

Acute infection of bees with IAPV causes a typical paralysis symptom. IAPV-infected bees start to die accompanied by body trembling at 36 h post-infection and paralytic bees are frequently observed at the same time. To further confirm and reflect the occurrence in the field, we collected bee samples with typical paralysis symptoms in the field and screened the nine most common bee viruses: IAPV, sacbrood virus (SBV), acute bee paralysis virus (ABPV), black queen cell virus (BQCV), chronic bee paralysis virus (CBPV), *Varroa destructor* virus (VDV), deformed wing virus (DWV), Chinese sacbrood virus (CSBV) and Kakugo virus (KV) (Table S1). Our results showed that IAPV was the predominant virus, which was in line with observations of bees infected by IAPV injection. To explore IAPV’s abundance in different tissues, we determined the viral load in seven tissues by quantifying the transcriptional levels using qPCR when bees exhibited the clinical symptoms. Compared to the control group (Figure 1A), our results revealed that the IAPV abundance in these tissues was different, ranging from 2.75 × 10^8^ to 1.1 × 10^3^ genomic copies in the following order from highest to lowest: trachea > fat body > hindgut > muscle > antenna > brain > midgut (Figure 1B). Similar results were obtained in bees with IAPV infection under natural conditions: trachea (2.73 × 10^9^ genome copies) > hindgut > antenna > fat body > midgut > muscle > brain (Figure 1C). These results indicated that regardless of injection or natural infection, IAPV was mainly distributed in the trachea, hindgut, antenna and fat body, followed by the midgut and muscle. Neither the midgut nor the fat body harbored the highest level of IAPV in *Apis mellifera*, and this was inconsistent with recent observations [10,14].

**Figure 2:**
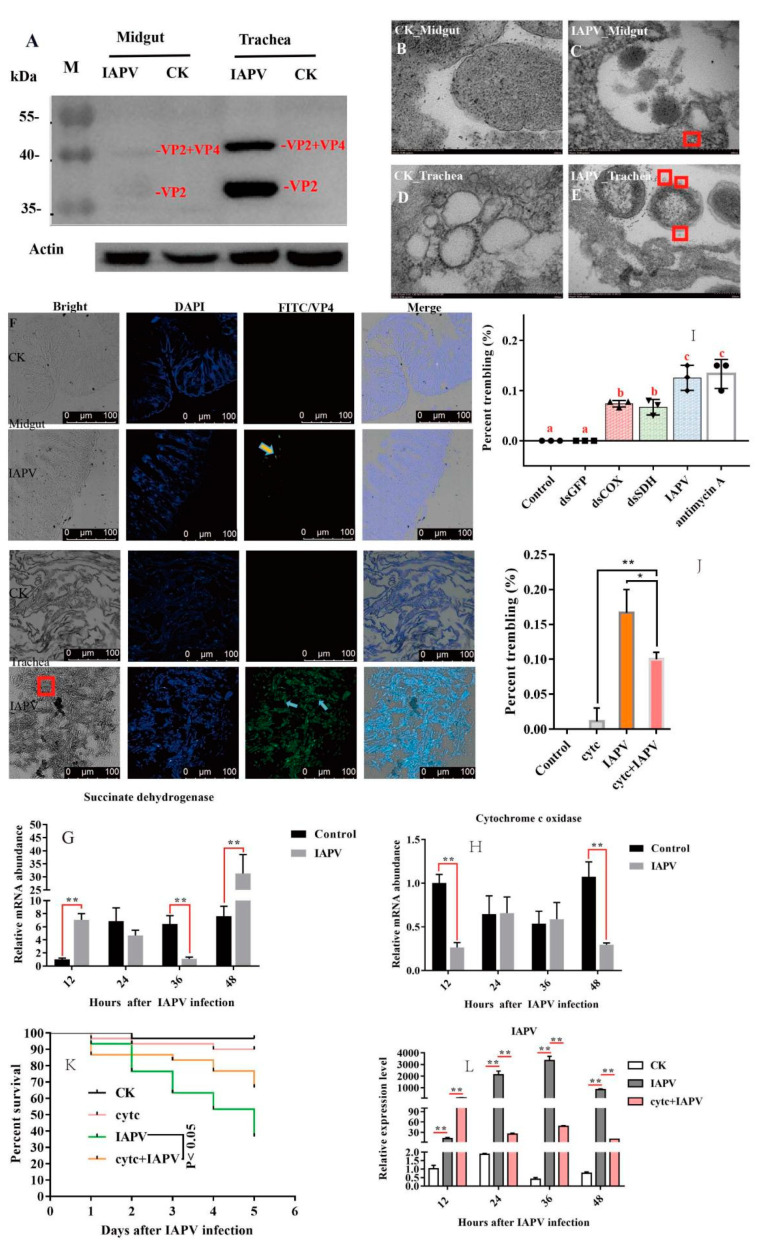
Tracheae harbored a higher level of IAPV and the effect of IAPV-induced paralytic symptoms associated with tachypnea in the tracheal system. Detection of the IAPV protein in midgut and tracheal system of bees by Western blotting with rabbit anti-VP2 polyclonal antibody at 96 h after IAPV infection, and the relative expression level of IAPV VP2 was normalized by actin using the ImageJ platform (**A**). The representative TEM images of the midgut section in control group (**B**) and IAPV infection group at 96 h after IAPV infection (**C**). The representative TEM images of the tracheal section in control group (**D**) and IAPV group at 96 h after IAPV infection (**E**). Red box indicates the viral particles of IAPV in the midgut and trachea. Localization of IAPV in trachea and midgut using anti-VP2 to detect VP2 (in green) at 96 h after IAPV infection (**F**). Red box indicates the damaged tracheal integrity. The impacts of IAPV infection on the expression of succinate dehydrogenase (**G**) and cytochrome c oxidase (**H**) genes related to respiratory system function at 12, 24, 36 and 48 h after IAPV infection. The number of bees with trembling symptoms after dsRNA treatment and respiratory chain inhibitor antimycin A (100 μM) treatment at 36 h after IAPV infection (**I**). The difference in the number of bees with trembling symptom treated with IAPV, cytochrome c (150 ng/bee) or IAPV and cytochrome c, an activator of the respiratory chain, at 36 h after IAPV infection (**J**). The survival rate of bees treated with IAPV, cytochrome c or IAPV and cytochrome c 5 days after IAPV infection (**K**). The IAPV relative expression level in bees treated with IAPV, cytochrome c or IAPV and cytochrome c at 12, 24, 36 and 48 h after infection with IAPV and treatment with cytochrome c (**L**). Asterisks indicate significant differences between groups (* *p* < 0.05, ** *p* < 0.01), and a, b and c represents a significant difference (*p* < 0.05) in subfigure (**I**).

To test the tissue damage caused by viral infection, histopathological analysis on the trachea, fat body, antenna, muscle, midgut and hindgut of honey bees was performed using a Leica DFC280 light microscope. IAPV infection resulted in varying degrees of damage in different tissues of bees both 36 h post-infection and in field-collected bees (Figure 1D–U). There was no significant difference in the antenna, muscle, midgut and hindgut between control and IAPV injection treatment groups, but trachea and fat body. Severe histopathologic changes were observed in these two tissues from both IAPV injection and natural infection groups (Figure 1M–R). Relative to control, tracheae from IAPV-injected and natural infection groups were darker, and the integrity of tracheae was completely destroyed in the IAPV natural infection group (Figure 1P–R). With both injection and natural infection, IAPV caused obvious heterogeneous cytoplasm of the fat body, and even vacuolization was found in the fat body cytoplasm of bees in the IAPV natural infection group (Figure 1O). A previous study showed that the gut harbored the highest level of IAPV among the tested tissues [10], but our Western blot results indicated that the expression level of IAPV protein was significantly higher in the trachea than in the midgut (*p* < 0.01) (Figure 2A). Next, we further examined the difference in the number of virus particles between the midgut and trachea. As expected, we found more virus particles in the trachea than in the midgut (Figure 2B–F). This suggested that IAPV caused the systemic infection pattern via turning the trachea into the preferred tissue tropism. This was in line with *Autographa californica* M nuclear polyhedrosis virus (AcMNPV) infection, which was disseminated within the different tissues of the host via tracheae [21].

To test the impacts of IAPV infection on tracheal system function, we measured the expression level of genes related to the respiratory chain. Subsequently, we found that the expression level of SDH was significantly down-regulated (*p* < 0.01) after IAPV infection at 36 h, compared to control groups (Figure 2G), while the expression of COX was significantly down-regulated (*p* < 0.01) at 48 h (Figure 2H). Next, to examine whether the trembling symptoms were due, in part, to the significant change in the two genes’ expression levels, we knocked down SDH and COX separately to observe the effect on paralytic symptoms for 36 h, and used antimycin A as a positive respiratory chain inhibitor [22]. Consistently, our results showed that dsRNA treatment caused significantly observable symptoms, e.g., body trembling, and we found similar effects with IAPV and antimycin A treatment groups, though the number of bees with trembling was smaller than those of IAPV and antimycin A-treated groups (Figure 2I). No trembling bees were observed in control groups (Movies 1–5). Since the dsRNA treatment for SDH and COX can aggravate the presence of typical paralysis symptoms, we tried to test whether a respiratory chain activator, cytochrome c (Cytc) [23], can mitigate the symptoms induced by IAPV. As expected, our results showed that 36 h after treatment with Cytc and IAPV, the number of bees with trembling was significantly smaller than that of the IAPV group (*p* < 0.01) (Figure 2J), and it remarkably extended the lifespan of bees relative to the IAPV-treated group (*p* < 0.05) (Figure 2K). This was in line with the expression level of IAPV that was significantly higher in IAPV-treated bees than those of Cytc- and IAPV-treated groups at 36 h post-infection (*p* < 0.01) (Figure 2L). It has been reported that Cytc can reduce *Bombyx mori* nucleopolyhedrovirus (BmNPV) infection [22]. Our results further confirmed that the trachea played a vital role in systemic infection of IAPV, which was similar to other insects infected by RNA viruses [24]. The trachea harbored the highest level of IAPV, and suggested that typical trembling was a “tachypnea” symptom induced by IAPV infection. In fact, acute cough, induced by tachypnea, as a major symptom of viral respiratory tract infection, is a physiological response to airway irritation and an important defense mechanism for the respiratory tract [25].

Our results have implications for understanding the relation between symptoms and determining factors as well as studying bee–virus interactions. The current study causes us to rethink the cause of typical symptoms of honey bee viruses. For example, typical DWV-induced symptoms, e.g., deformed wings, do not mean that the wings of bees infected with DWV harbor the highest titers of DWV [26]. In addition, our results lay the groundwork for molecular mechanisms of viral infection and transmission routes, especially for exploring the underlying mechanism of typical symptoms of honey bee viruses, and also provide a path forward to study the interaction between viral infection and immune response at the tissue level.

## 3. Materials and Methods

### 3.1. Samples

The brood frames from three different colonies, collected from the colonies of experimental apiaries of the Institute of Apicultural Research (IAR), Chinese Academy of Agricultural Sciences (Beijing, China), were transferred to an incubator (30 ± 1 °C, 60% relative humidity (RH)) for emergence. Newly emerged honey bees (*Apis mellifera*) were collected within 24 h, and screened for the presence of common honey bee viruses, including IAPV, sacbrood virus (SBV), deformed wing virus (DWV), chronic bee paralysis virus (CBPV), Varroa destructor virus (VDV), Chinese sacbrood virus (CSBV), black queen cell virus (BQCV), acute bee paralysis virus (ABPV) and Kakugo virus (KV) (Table S1) (as previously described [27,28]). About 30 newly emerged bees were transferred to a wooden container and kept in an artificial climate incubator (MGC-800HP, Shanghai, China), and 2 mL 50% sucrose was provided every day if they were negative for the bee viruses mentioned above. To further confirm and reflect the occurrence in the field, we collected bee samples with typical paralysis symptoms in the field and screened for the 9 common bee viruses (Figure S1).

### 3.2. Construction of IAPV Infectious Clone

Constructing the infectious clone of IAPV and RNA synthesis were performed as previously described [29]. In brief, the full-length genome of IAPV was amplified from bee samples collected in our experimental apiaries using RT-PCR. Three fragments of IAPV were amplified with high-fidelity Phusion HiFi PCR Master Mix (NEB, Ipswich, MA, USA) using specific primers [29]. A low copy vector, pACYC177 (CWBio, Beijing, China), was used to generate a stable clone. The full-length infectious clone (pACYC177-IAPV) was completed using homologous recombination according to the manufacturer’s instructions with a ClonExpressMultiS One-Step Cloning Kit (Vazyme Biotech Co., Nanjing, China). For RNA synthesis in vitro, the plasmid pACYC177-IAPV was used as the template to amplify the full-length IAPV by high-fidelity Phusion HiFi PCR Master Mix (NEB, Ipswich, MA, USA) and the RNA was purified with an EasyPure RNA Kit (TransGen Biotech, Beijing, China). Newly emerged bees were confirmed to be negative for common viruses, and then used for an infection experiment with three repetitions for each treatment. Thirty bees were injected with 2 µL of purified synthetic RNA (approximately 1 × 10^12^ genome copies) into the third to fourth integument of the abdomen with a Hamilton syringe (702) (Hamilton, Bonaduz, Switzerland). The control group was injected with PBS. Then, these groups were transferred to an incubator at 30 °C/60% RH. The infected and healthy bees were observed and collected every 12 h, respectively. The mortality was observed and recorded daily.

### 3.3. RNA Extraction and qPCR Assays

Five bees were collected from each group, and total RNAs from the trachea, fat body, hindgut, muscle, antenna, brain, midgut and leg were extracted with an RNAprep Pure Micro Kit (TIANGEN, Beijing, China) according to the manufacturer’s instructions. About 500 ng RNA for each sample with A260/280 ratios ranging from 1.8 to 2.2 were used for cDNA synthesis with the PrimeScript RT Reagent Kit with gDNA Eraser (TaKaRa, Dalian, China) according to the manufacturer’s instructions. The cDNAs of all samples were stored at −20 °C until use.

The cDNAs of all samples were used as the template to perform qPCR on an Applied Biosystems 9600 Real-Time PCR system (Bioer, Hangzhou, China). The amplification efficiencies ranged from 92 to 107% and correlation coefficients (R^2^) were greater than 0.99, which fit the RT-qPCR requirements [30]. The qPCR reaction system consisted of 12.5 μL 2 × SYBR Premix Ex TaqTM II (Takara, Dalian, China), 0.5 μL each of the forward and reverse primers (10 mM), 2 μL cDNA template and 9.5 μL double-distilled H_2_O for a total volume of 25 μL. qPCR was performed using a cycling profile of an initial cycle at 95 °C for 30 s; 40 cycles of 95 °C for 5 s for denaturation, 25 s at 55 °C for annealing and 20 s at 72 °C for extension and the generation of a melting curve consisting of a single peak to rule out nonspecific products and primer dimers afterwards. The qPCR analysis was performed with three biological replicates for each sample. The specific primers for qPCR are shown in Table S2.

### 3.4. Tissue Dissection and Histopathological Examination

To verify IAPV proliferation in different tissues, five bees were collected from each of three groups maintained in an incubator at 12, 24, 36 and 48 h post-infection with IAPV. Bee samples were fixed on the wax top of a dissecting dish with insect pins under a dissecting microscope, and then were cut along the dorsal midline with scissors from the tip of the abdomen to the head. The scissors and forceps were washed three times with 0.3% sodium hypochlorite followed by a final rinse in sterile water after isolating each tissue. Tissues, including the trachea, fat body, hindgut, muscle, antenna, brain, midgut and leg, were carefully removed from each bee. Then, the dissected tissues were washed in 1 × PBS buffer (10 mM, pH 7.4). In addition, bees with obvious paralytic symptoms of IAPV were collected in the field and subjected to tissue dissection.

To study the damage caused by virus infection to various tissues, histopathological analysis on the trachea, muscle, midgut, fat body, antenna and hindgut of honey bees was performed using a Leica DFC280 light microscope as described previously by Fischer et al. [31]. Briefly, these eight tissues were taken from five bees at 36 h after IAPV infection, which were from each of the three colonies maintained in an incubator, and then quickly fixed with 4% paraformaldehyde and stored at 4 °C. Next, conventional dehydration followed by paraffin embedding was performed and then the slides with tissues were dewaxed in dimethylbenzene and rehydrated in alcohol of diminishing concentrations. Subsequently, hematoxylin and eosin (HE) staining were performed to examine the pathological changes in various tissues under a Leica DFC280 light microscope and analyzed using the Leica Q Win Plus V3 Image Analysis System (Leica Micros Imaging Solutions Ltd.; Cambridge, UK). Similarly, tissues from field-collected bees infected with IAPV under natural conditions were dissected.

### 3.5. Western Blot and Immunofluorescence Analysis

To determine the difference in IAPV titer between the trachea and midgut, five individual bees infected with IAPV after 96 h were obtained from each of the three colonies. Protein was extracted from the midgut and trachea of bees using RIPA lysis buffer (Lablead, Beijing, China) according to the manufacturer’s instructions. Briefly, the midgut and trachea were ground with steel beads in 200 µL RIPA lysis buffer containing 0.2% DTT and 1 µM PMSF. Then, the mixture was centrifuged at 12,500 rpm for 10 min at 15 °C. A 100 µL supernatant protein sample was added to 20 μL of 5 × SDS-PAGE buffer (CWBIO, Shanghai, China), boiled for 3 min and subjected to SDS-PAGE at a constant voltage (100 V). The polypeptides were separated on 12% SDS-PAGE prior to electrophoretic transfer to nitrocellulose membranes (Pall Corporation, New York, NY, USA). Then, the gels were fixed in 0.85% o-phosphoric acid/20% methanol for 30 min, and then stained overnight in Coomassie Brilliant Blue R250 (ZOMANBIO, Beijing, China) according to the manufacturer’s instructions. Subsequently, the gels were destained in 25% methanol and the protein was transferred to a nitrocellulose membrane (PVDF) (Bio-Rad, Hercules, CA, USA). The blots were blocked with 5% skim milk powder and probed with different rabbit polyclonal antibodies against IAPV VP2 synthesized by Hangzhou HuaanBiotech (Hangzhou, China). Next, the blots were detected using Amersham ECL plus reagent (GE Healthcare, Chalfont St Giles, GB, UK) and exposed to a C-Digit scanner (Licor, Lincoln, NE, USA) and imaged by photography.

The midgut and trachea of honey bees were dissected and removed as mentioned above and then were fixed with 4% formaldehyde for 60 min, followed by freezing with a tissue embedding agent (Sakura Finetek, Japan) at −20 °C. Subsequently, 5 µm frozen sections of midgut and trachea were cut and affixed onto glass slides to enhance tissue adhesion (Platinum pro; Matsunami Glass Ind., Kishiwada, Japan). Slides were washed for 10 min with distilled water and PBS (pH 7.4), respectively. To maintain the autofluorescence intensity, we recycled the antigen to enhance the intensity of the primary antibody binding. Briefly, we boiled slides with target tissues for 3 min in 0.1 M citrate buffer, and then they were allowed to spontaneously cool to room temperature under the same buffer. Next, the sections were soaked in washing buffer (0.1% Tween 20 in PBS) for 15 min and slides were blocked for 1 h with PBS buffer containing 5% milk at room temperature. The primary antibodies (against IAPV VP2 from rabbit, dilution, 1:1000) were diluted in block and incubated with the sections overnight at 4 °C. Then, these sections were washed three times in PBS and incubated with fluorescein isothiocyanate (FITC)-conjugated goat anti-rabbit secondary antibody (AlexaFluor488, Invitrogen, Waltham, MA, USA) (dilution, 1:200). After rinsing three times in 0.01M PBS, the nuclei were stained with 4′,6-diamidino-2-phenylindole (DAPI) (Roche) for 4 min at room temperature and observed with laser scanning confocal microscopy (TCS SP8 STED, Leica, German). The stained midgut and trachea were analyzed using confocal laser scanning microscopy (FluoView FV1000; Olympus, Tokyo, Japan) with automated image analysis software. Experiments were performed three times.

### 3.6. Transmission Electron Microscopy

Five honey bees were collected from each of the three colonies maintained in an incubator at 96 h after IAPV infection. The midgut and trachea were dissected and removed as mentioned above, and were prepared for transmission electron microscopy (TEM) as published previously by Pilgrim et al. [32]. In brief, tissues were dissected into 2% (wt/vol) paraformaldehyde containing 2.5% (wt/vol) glutaraldehyde in 0.1 M phosphate buffer (pH 7.4), which was as fixative. Then, heavy metal staining consisting of ddH_2_O was performed using 2% (wt/vol) OsO4, followed by 1% (wt/vol) tannic acid and then 1% (wt/vol) aqueous uranyl acetate. To prevent precipitation, washing the tissue with ddH2O between each staining step was carried out. A Pelco BiowavePro (Ted Pella Inc., Redding, CA, USA) was used to perform fixation and staining steps at 100 W 20 Hg, for 3 min and 1 min, respectively. Dehydration was in a graded ethanol series before filtration and embedding in medium premix resin (TAAB, Reading, UK). For TEM, the tissues were cut into 70 to 74 nm serial sections and collected on Formvar-coated (0.25% (wt/vol) in chloroform; TAAB, Reading, UK) Gilder 200 mesh copper grids (GG017/C, TAAB, Reading, UK) using a UC6 ultra microtome (Leica Microsystems, Wetzlar, Germany). Images were acquired on a 120 kV HT7700 (HITACHI, Tokyo, Japan).

### 3.7. RT-qPCR Assays on Genes Related to the Respiratory System

To test the effect of IAPV infection on the expression level of genes related to the respiratory system, 5 honey bees were collected from each of the three colonies at 12, 24, 36 and 48 h after IAPV infection. Based on the results above about impairment in the trachea, we assessed whether the IAPV infection can induce the alteration of the expression level of genes related to respiration, and then we selected the eight genes related to the respiratory chain, including NADH-coenzyme Q reductase, cytochrome c oxidase (COX), iron–sulfur protein NUBPL, cytochrome b-c1 complex subunit Rieske, NADH-ubiquinone oxidoreductase, electron transfer flavoprotein–ubiquinone oxidoreductase, ATP synthase and succinate dehydrogenase (SDH). The specific primers for these eight genes are shown in Table S2. Translation initiation factor eIF2B was used as a reference gene and three technical replicates were performed as described by Deng et al. [31]. As shown in S3, we speculated that IAPV infection disrupts the expression of genes of the respiratory electron transport chain.

### 3.8. Preparation of dsRNA and Assessment of the Knockdown Efficiencies by Assay of Enzyme Activity

As shown in Figure S4, only SDH and COX genes were down-regulated when IAPV proliferated. To clearly understand the cause and effect between paralytic symptoms and respiratory failure, we quantified the expression levels of target genes from bees treated with dsRNA. dsRNAs were generated as previously described [33]. Briefly, the preparation of template DNA of two respiratory chain genes (SDH and COX) and GFP was performed with gene-specific amplicons, approximately 300–500 bp in length. Gene-specific primers for them and the dsGFP gene were designed, including T7 promotor sequences (TAATACGACTCACTATAGGGAGA) at the 5′ ends of each strand, and were subsequently used to amplify target fragments using PCR from cDNA (Table S3). The reaction products were digested using DNase and RNAse at 37 °C for 1 h. Next, we generated RNA strands from the transcript using MEGAscript RNAi Kit (Invitrogen, USA) at 37 °C for 4 h in vitro. dsRNA was purified with RNAeasy and quantified with a spectrophotometer (NanoDrop Technologies, Wilmington, NC, USA). Then, 30 newly emerged bees in each group were confirmed to be negative for other common viruses, and then used for further experiments with three repetitions for each treatment. Five micrograms of dsRNA of COX and SDH and GFP was injected into newly emerged *A. mellifera*, and no treatment was given for the control group. After injection dsRNA, 10 bee tracheae in each group were collected to detect the expression level and the activity of COX and SDH. The dead and paralyzed bees with obvious trembling were observed, photographed and recorded daily. To detect the knockdown efficiencies at the protein level, we tested the activity of succinate dehydrogenase (SDH, U/g and cytochrome c oxidase (COX, U/mg) of tracheae, and the total protein concentration was determined using the BCA Protein Assay Kit (CWBIO, Shanghai, China) according to the instructions. The SDH Activity Test Kit and COX Activity Assay Kit were supplied by Solarbio (Beijing, China) and Nanjing Jiancheng Bioengineering Institute (Nanjing, China), respectively. Briefly, for the measurement of SDH activity, 10 bee tracheae were ground, lysed and centrifuged at 11,000× *g* for 10 min at 4 °C. At the same time, a reaction mixture containing 60 μL phenazine dimethyl sulfuric acid, 60 μL succinate and 800 μL double-distilled water was incubate at 25 °C for 10 min. After that, 30 μL lysed supernatant total protein (about 10 μg/μL) and 30 μL 2,6-dichlorophenol indophenol were added to the 920 μL reaction mixture to a final volume of 1 mL and the absorbance was measured at 600 nm at 25 °C per min. Then, enzyme activity was calculated according to the total protein concentration and change in absorbance within 1 min. For measurement of COX activity, 10 bee tracheae were ground and lysed by special lysis buffer at 4 °C for 30 min, and centrifuged at 16,000× *g* for 5 min at 4 °C. Next, a reaction mixture containing reduced cytochrome c, special buffer and 100 μL lysed supernatant total protein (about 3 μg/μL) in a final volume of 1 mL was incubated at 25 °C for 3 min. After that, the absorbance was measured at 550 nm at 25 °C per min. Finally, enzyme activity was calculated according to the supernatant total protein concentration and change in absorbance within 1 min. All the experiments were performed in three biological replicates.

### 3.9. Identification the Relation between Paralysis and Respiratory

To further confirm the links between typical symptoms and respiratory failure, we used antimycin A and cytochrome c as a positive inhibitor and activator, respectively. About 30 newly emerged bees in each group were collected and transferred to a wooden container and kept in an artificial climate incubator (MGC-800HP, Shanghai, China), 2 mL 50% sucrose with 100 μM antimycin A was provided every day and then bees with paralysis symptoms were recorded. Bees were fed with 50% sucrose as a negative control.

To test the effect of the respiratory chain activator cytochrome c on paralysis symptoms, we measured the IAPV proliferation after treatment with cytochrome c. About 30 newly emerged bees were transferred to a wooden container and kept in an artificial climate incubator, with three repetitions for each treatment. Treated bees were injected with 2 µL of purified synthetic RNA (approximately 1 × 10^12^ genome copies) into the third to fourth integument of the abdomen with a Hamilton syringe (702) (Hamilton, Bonaduz, Switzerland), while the control group was injected with PBS. Ten hours after injection, these bees were injected with 1 µL cytochrome c (150 ng/bee), then transferred to an incubator at 30 °C and 60% relative humidity and mortality was observed and recorded daily. Then, tracheae of 5 bees in each group were collected to detect IAPV proliferation at 12, 24, 36, 48 h. In addition, 5 tracheae in every group were collected to detect the activity of succinate dehydrogenase and COX 48 h after injection of IAPV. Antimycin A and cytochrome c were supplied by Sigma-Aldrich (St. Louis, MO, USA) and Solarbio (Beijing, China), respectively.

### 3.10. Statistical Analysis

For measuring the IAPV titer by qPCR, data were shown as mean ± SEM, and the variation between the treatments was analyzed using one-way ANOVA. The 2^−∆∆CT^ method was employed to validate and quantify the expression levels of genes related to respiration. One-way ANOVA was used to analyze the variance and the significant variations were analyzed by Tukey’s multiple comparison test. For IAPV infection assays, bees that died before measurement were excluded from the analysis. The average numbers of trembling bees among the different treatment groups was shown as mean ± SEM and Tukey’s multiple comparison was conducted to detect any significant variation among replicates. All analyses were performed using GraphPad Prism statistical software (GraphPad Prism 7.0). The grayscale of Western blot bands and the fluorescence intensity of immunofluorescence between the IAPV treatment and control groups were measured using grayscale image analysis on the ImageJ platform (version 1.52, Bethesda, MD, USA). The values of grayscale and fluorescence intensity among the different groups were calculated and the significant variations were analyzed by Tukey’s multiple comparison test. *p* < 0.05 (*) and *p* < 0.01 (**) represent significantly and extremely different values at the 0.05 and 0.01 levels.

## Data Availability

Not applicable.

## Supplementary Materials

Supplementary materials can be found at https://www.mdpi.com/article/10.3390/ijms221810078/s1.
