# Supplementary material for: IAPV-Induced Paralytic Symptoms Associated with Tachypnea via Impaired Tracheal System Function
Source: Int J Mol Sci. 2021 Sep 17;22(18):10078. doi: 10.3390/ijms221810078 (PMC8469059; doi:10.3390/ijms221810078)
Supplement: Supplementary file 1 [file ijms-22-10078-s001.zip › ijms-1343793-supplementary.pdf]

International Journal of *Molecular Sciences*

Supplementary Information for

IAPV-Induced Paralytic Symptoms Associated with Tachypnea via Impaired Tracheal System Function

Yanchun Deng [1,2,3,†], Sa Yang [1,2,†], Hongxia Zhao [4], Qingyun Diao [1,2] and Chunsheng Hou [1,2,*]

SI Figures

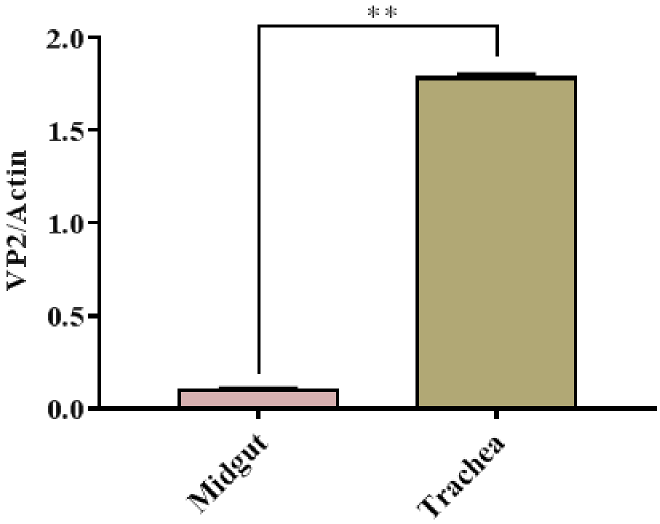

Figure S1. The relative expression level of IAPV VP2 by against actin.

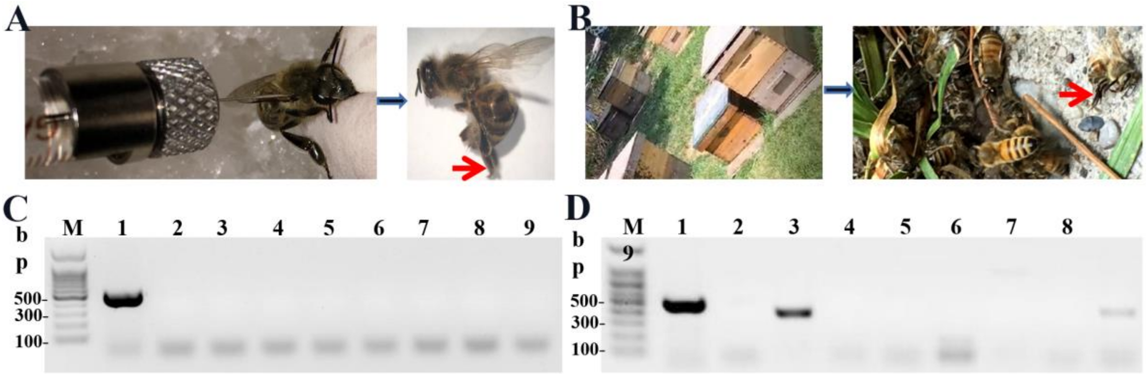

Figure S2. Detection of the presence of IAPV in bees infected with infectious clone and under natural conditions.(**A**) The way to infect bees with IAPV infectious clone and caused the typical symptom (red arrow). Bees with typical symptom were collected in field (red arrow) (**B**). The detection of 9 common viruses on bees after 48 hours IAPV infection (**C**) and bees from field-collected (**D**) using RT - PCR. 1: IAPV; 2: Sacbrood virus (SBV); 3: Deformed wing virus-A (DWV); 4: Varroa destructor virus (VDV); 5: Chronic bee paralysis virus (CBPV); 6: Chinese sacbrood virus (CSBV); 7: Acute bee paralysis virus (ABPV); 8: Black queen cell virus (BQCV); 9: Kakugo virus (KV).

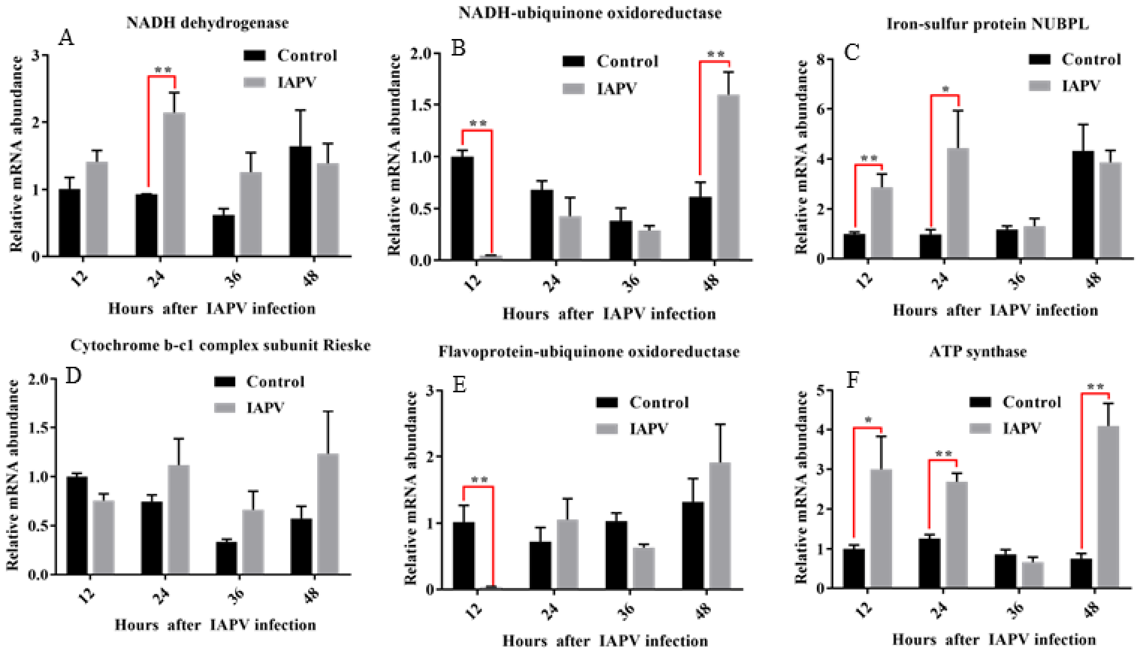

Figure S3. The expression level of respiratory system related genes in bees after IAPV infection. The expression levels of detected genes for *NADH dehydrogenase* (**A**), *NADH-ubiquinone oxidoreductase* (**B**), *Iron-sulfur protein NUBPL* (**C**), *Cytobrome b-c1 complex subunit rieske* (**D**), *Flavoprotein-ubiquinone oxidoreductase* (**E**) and *ATP synthase* (**F**).

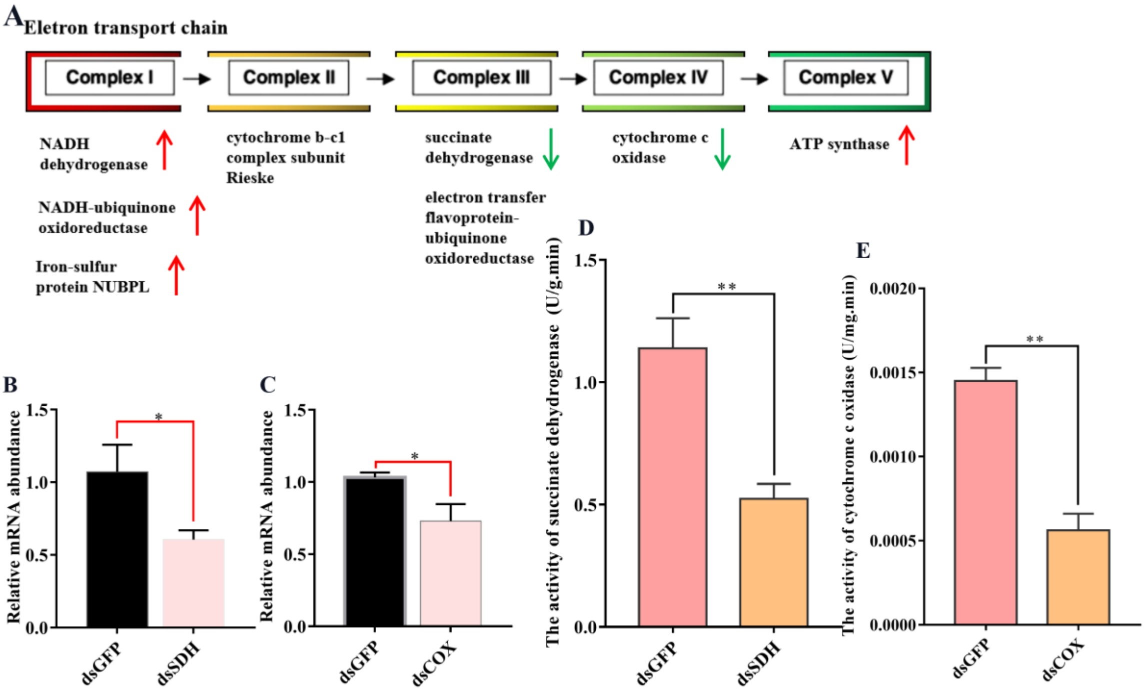

Figure S4. Knockdown efficiencies. (**A**) The schematic diagram of respiratory chain. Red indicated the up-regulated genes and blue down-regulated genes. The expression levels of *succinate dehydrogenase* (SDH) (**B**) and *cytochrome c oxidase* (COX) (**C**) gene in bees trachea after RNA interference. (**D**) The activity of succinate dehydrogenase (U/g.min) of trachea in bees at 36 hours after dsSDH (5 μg/bee) treatment. (**E**) The activity of cytochrome c oxidase (U/mg.min) of trachea in bees at 36 hours after dsCOX (5 μg/bee) treatment. Asterisks indicate significant differences of respective points ($*P < 0.05$, $**P < 0.01$).

SI Tables

Table S1. Primers used for PCR detection common honey bee viruses.

Virus	Primer（5′-3′）	Annealing Temperature (°C)	Target Fragment (bp)
IAPV	F:AGACACCAATCACGGACCTCAC<br>R:AGATTTGTCTGTCTCCCAGTGCACT	55	475
SBV	F:ATATACGGTGCGAGAACTGC<br>R:CTCGGTAATAACGCCACTGT	56	879
ABPV	F:TTATGTGTCAGAGACTGTAT<br>R:GCTCCTATTGCTCGGTTTTTC	55	900
BQCV	F:TGGTCAGCTCCCACTACCTTAAAC<br>R:GCAACAAGAAGAAACGTAAACCAC	57	700
CBPV	F:TCAGACACCGAATCTGATTATTG<br>R:ACTACTAGAAACTCGTCGCTTCG	55	570
VDV	F:CATAGCGAATTACGGTGCAA<br>R:GAGGGGTCCCTACTCTACCG	55	200
DWV	F:CTTACTCTGCCGTCGCCCA<br>R:CCGTTAGGAACTCATTATCGCG	55	376
CSBV	F:CCTGGGAAGTTTGCTAGTATTTACG<br>R:CCTATCACATCCATCTGGGTCAG	55	161
KV	F:GATATGACTGTATCCTCCATAGCATCTC<br>R:GTATGAAACATATGGCACCTCAAAAGTA	55	396

Table S2. Primers used for Real-time Fluorescent Quantitative PCR.

Gene name	Primer Sequence (5′–3′)	GenBank Accession No.	Amplicon Size (bp)
IAPV	F:CCAGCCGTGAAACATGTTCTTACC<br>R:ACATAGTTGCACGCCAATACGAGAAC	KX421583.1	167
Cytochrome b-c1 complex subunit Rieske	F:GTGACCGGAAGGGGTATTGT<br>R:AACCTCCTCTGCGTTGCTAA	XM_006557503.3	155
NADH-ubiquinone oxidoreductase	F:TCGAGAAATATTGCGATGAATCGAG<br>R:ACTGAAAGCGCACTCCTCTC	XM_397330.6	213
electron transfer flavoprotein-ubiquinone oxidoreductase	F：GTCGATTTTGTCCTGCTGGTG<br>R：CCCACCTTCTGGTACAACCC	XM_624719.6	155
NADH dehydrogenase	F: ATCGTCCATTTTAGGTTCGC<br>R: TCTGCCATTCTGTAATCCCGT	NM_001172404.1	143
Cytochrome c oxidase	F:TCGTTTCGTACTTTGTACGTCTG<br>R: TGCTCGAACAATGCCATCTT	XM_006567340.3	108
Iron-sulfur protein NUBPL	F: ATTTGGTCCTTCTGTGCCGT<br>R: CAACGGACCCCATGCTACTT	XM_006571868.3	208
Succinate dehydrogenase	F:TTGGCAGCACTGGTATTCATCA<br>R:CGTCGTTGTGGGAGGATTTC	XM_623062.6	185
ATP synthase	F:CTGGCTGCTTGCACATATTAAG<br>R:CATCGCTAACTGGTTTATCAGCA	XM_003250744.4	110

Table S3. Primer pairs used for dsCOX, dsSUCC and dsGFP.

Genes	Primer sequence1 (5′–3′)	Amplicon size (bp)	GenBank accession no.
Cytochrome c oxidase	F:TAATACGACTCACTATAGGGAGAGGAGCG GTTTCGGTATTTTGG<br>R:TAATACGACTCACTATAGGGAGAAAAGCA GTTTCTCCGGGCAT	250	XM_006567340.3
Succinate dehydrogenase	F:TAATACGACTCACTATAGGGAGAAGCAGG ATTACGTGCAGCTT<br>R:TAATACGACTCACTATAGGGAGAGAGTGA CCTGTCCTGTCTGC	384	XM_623062.6
GFP	F:TAATACGACTCACTATAGGGAGATTCATGG CCAACACT TGTCC<br>R:TAATACGACTCACTATAGGGAGACAAGAA GGACCATGTGGTC	268	U17997; Clontech

Video S1-S5. The relationship between typical paralysis symptoms and respiratory failure in bee treated without (S1) and with dsRNA-SDH (S2), dsRNA-COX (S3), IAPV (S4) and antimycin A (S5).
